# Intratumoral and peritumoral radiomics for the pretreatment prediction of pathological complete response to neoadjuvant chemotherapy based on breast DCE-MRI

**DOI:** 10.1186/s13058-017-0846-1

**Published:** 2017-05-18

**Authors:** Nathaniel M. Braman, Maryam Etesami, Prateek Prasanna, Christina Dubchuk, Hannah Gilmore, Pallavi Tiwari, Donna Pletcha, Anant Madabhushi

**Affiliations:** 1 Department of Biomedical Engineering, Case Western Reserve University, Cleveland, OH 44106 USA; 2 University Hospitals Case Medical Center, Cleveland, OH 44106 USA

**Keywords:** Imaging, MRI, Neoadjuvant chemotherapy, Treatment response, Radiomics, Personalized medicine

## Abstract

**Background:**

In this study, we evaluated the ability of radiomic textural analysis of intratumoral and peritumoral regions on pretreatment breast cancer dynamic contrast-enhanced magnetic resonance imaging (DCE-MRI) to predict pathological complete response (pCR) to neoadjuvant chemotherapy (NAC).

**Methods:**

A total of 117 patients who had received NAC were retrospectively analyzed. Within the intratumoral and peritumoral regions of T1-weighted contrast-enhanced MRI scans, a total of 99 radiomic textural features were computed at multiple phases. Feature selection was used to identify a set of top pCR-associated features from within a training set (*n* = 78), which were then used to train multiple machine learning classifiers to predict the likelihood of pCR for a given patient. Classifiers were then independently tested on 39 patients. Experiments were repeated separately among hormone receptor-positive and human epidermal growth factor receptor 2-negative (HR^+^, HER2^−^) and triple-negative or HER2^+^ (TN/HER2^+^) tumors via threefold cross-validation to determine whether receptor status-specific analysis could improve classification performance.

**Results:**

Among all patients, a combined intratumoral and peritumoral radiomic feature set yielded a maximum AUC of 0.78 ± 0.030 within the training set and 0.74 within the independent testing set using a diagonal linear discriminant analysis (DLDA) classifier. Receptor status-specific feature discovery and classification enabled improved prediction of pCR, yielding maximum AUCs of 0.83 ± 0.025 within the HR^+^, HER2^−^ group using DLDA and 0.93 ± 0.018 within the TN/HER2^+^ group using a naive Bayes classifier. In HR^+^, HER2^−^ breast cancers, non-pCR was characterized by elevated peritumoral heterogeneity during initial contrast enhancement. However, TN/HER2^+^ tumors were best characterized by a speckled enhancement pattern within the peritumoral region of nonresponders. Radiomic features were found to strongly predict pCR independent of choice of classifier, suggesting their robustness as response predictors.

**Conclusions:**

Through a combined intratumoral and peritumoral radiomics approach, we could successfully predict pCR to NAC from pretreatment breast DCE-MRI, both with and without a priori knowledge of receptor status. Further, our findings suggest that the radiomic features most predictive of response vary across different receptor subtypes.

**Electronic supplementary material:**

The online version of this article (doi:10.1186/s13058-017-0846-1) contains supplementary material, which is available to authorized users.

## Background

For the 10% to 20% of the 230,000 patients diagnosed with breast cancer each year [1] who have locally advanced breast cancer, it is imperative to receive effective treatment as quickly as possible. These patients sit at a critical clinical juncture: Their tumor has spread beyond the breast to the chest wall, skin, or lymph nodes but has not yet metastasized further. Even with treatment, most patients with locally advanced breast cancer will develop distant metastases [2]. Neoadjuvant chemotherapy (NAC) is often a first line of defense in the treatment of locally advanced breast cancer. Administered prior to surgery, NAC can reduce tumor extent, increase a patient’s surgical options, and reduce metastasis [3]. The ideal outcome of NAC is the complete absence of residual invasive tumor cells within excised breast tissue following NAC, or pathological complete response (pCR), which strongly predicts favorable prognosis as compared with patients who experience partial or no response (non-pCR) [4, 5]. Less than 10–50% of breast cancer patients who undergo NAC achieve pCR, depending on receptor status subtype [6], and thus there is a need for reliable noninvasive pretreatment predictors of pCR that can enable better targeting of NAC and prevent a delay in effective treatment for patients with nonresponding or progressive tumors.

Because of its high sensitivity to tumor presence and angiogenic changes, dynamic contrast-enhanced magnetic resonance imaging (DCE-MRI) is the preferred imaging modality in the NAC setting and has been demonstrated to effectively predict pCR following an early treatment period. For instance, changes in volumetric and kinetic parameters perform well in pCR prediction but afford no intuition with regard to pCR prior to treatment [7–10]. There remains a shortage of reliable clinical pCR indicators based on DCE-MRI that do not require previous NAC administration [7, 10].

Radiomic textural analysis—the high-throughput extraction of quantitative imaging features characterizing the spatial relationships and consistency of signal intensities—within the tumor region has been shown to enable pCR prediction across a range of cancer types and imaging modalities [11]. Many of these features, such as Haralick gray-level co-occurrence matrix-based features, quantify enhancement heterogeneity, which has been shown to predict aggressive growth, unfavorable prognosis, and poor treatment response in breast cancer [12–19]. Within the realm of breast cancer, DCE-MRI radiomics has been shown to be effective in predicting breast cancer biology, including receptor status [15, 20, 21], subtype [13, 20–23], and genomics [12, 24, 25].

Previous work exploring radiomics within the whole breast parenchyma has suggested the importance of studying the tumor’s physiological environment in building predictive models of tumor response and outcome [22, 26–28]. However, evidence suggests that the peritumoral region—the area immediately surrounding the tumor mass—may possess valuable outcome-related information that is not effectively captured by radiomic analysis of the bulk parenchyma. Angiogenic [29] and lymphangiogenic [30] activity, as well as peritumoral invasion of lymphatics and blood vessels [31, 32], is a predictor of survival that manifests within the peritumoral region on imaging studies. Signatures of immune response present within peripheral breast tissue, such as stromal response [33] and peritumoral lymphocytic infiltration [34], also have documented correlations with outcome. Therefore, intratumoral and parenchymal radiomics may be missing crucial markers of response located in the surrounding tumor microenvironment.

In this study, we explored the application of intratumoral and peritumoral radiomics for the noninvasive prediction of pCR in NAC recipients based on pretreatment DCE-MRI. We identified a radiomic profile of response consisting of the intratumoral and peritumoral features that best collectively distinguishes pCR and non-pCR. We assessed the added value of peritumoral radiomics and used our radiomic profile to predict pCR. In the second half of this study, we divided our dataset by receptor type and again identified radiomic profiles of response specific to receptor subgroup. We then investigated whether a subtype-specific profile of intratumoral and peritumoral radiomics enhances the ability to predict response.

## Methods

**Fig. 1:**
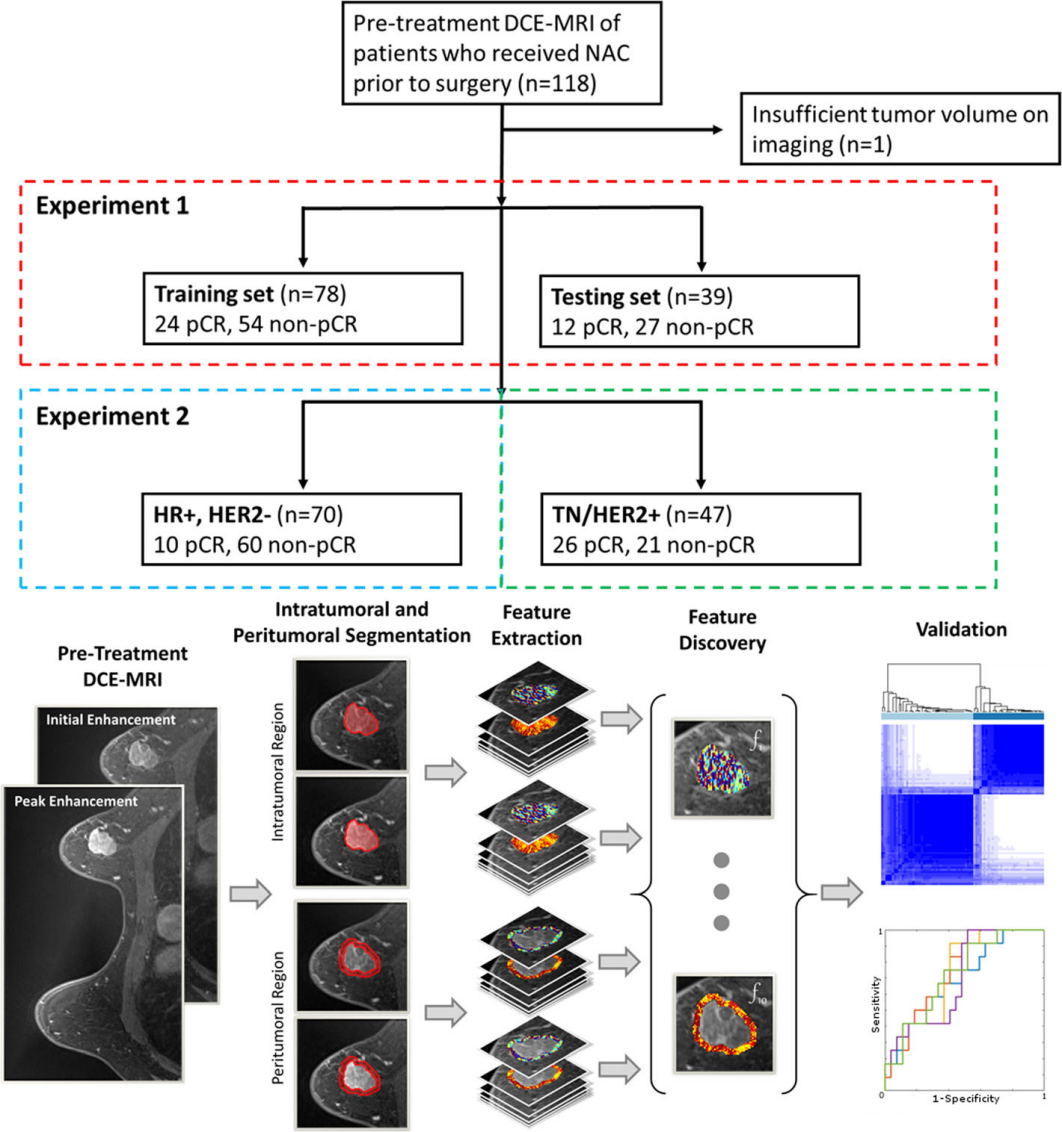
*Top*: Patient selection flowchart for experiments 1–3. *Bottom*: Radiomic pathological complete response (pCR) prediction pipeline. *DCE-MRI* Dynamic contrast-enhanced magnetic resonance imaging, *ER* Estrogen receptor, *HER2* Human epidermal growth factor receptor 2, *HR* Hormone receptor, *NAC* Neoadjuvant chemotherapy, *TN* Triple-negative

This Health Insurance Portability and Accountability Act of 1996 (“HIPAA”) regulations-compliant study was approved by institutional review board at the University Hospitals Case Medical Center, and the need for informed consent was waived. Figure 1 depicts patient selection and overall experimental design. All 118 patients with biopsy-proven primary breast cancer who received NAC prior to breast surgery and had breast DCE-MRI before initiation of NAC at the Cleveland University Hospitals network between 1 March 2012 and 15 May 2016 were retrospectively included in this study. One patient was eliminated from the dataset for insufficient tumor volume on imaging, leaving a dataset of 117 patients (Table 1). Human epidermal growth factor receptor 2-negative (HER2^−^) patients received doxorubicin and cyclophosphamide every 2 weeks for four cycles followed by four cycles of paclitaxel. All HER2^+^ patients received treatment with docetaxel and trastuzumab every 3 weeks for six cycles. The HER2^+^ treatment regimen was also supplemented with pertuzumab and/or carboplatin on a patient-by-patient basis, depending on disease severity and availability at the time of treatment.

**Table 1:** Breakdown of dataset by pathological complete response status

	pCR	Non-pCR
Number of patients	36	81
Mean age, years	53 (23–72)	48 (26–79)
Receptor status		
ER^+^	17	66
PR^+^	15	58
HER2^+^	16	12
TN	10	9
1.5-T DCE-MRI	30	60
Scanner 1	17	40
Scanner 2	12	15
Scanner 3	1	5
3-T DCE-MRI	6	21
Scanner 4	0	2
Scanner 5	3	10
Scanner 6	3	9
Lesion diameter, cm		
Mean	3.9	4.2
SD	2.4	2.6
Stage		
I	5	6
II	18	43
III	13	31
IV	0	1
Enhancing mass	29	69
Non-mass enhancement	7	12

*Abbreviations: DCE-MRI* Dynamic contrast-enhanced magnetic resonance imaging, *ER* Estrogen receptor, *HER2* Human epidermal growth factor receptor 2, *pCR* Pathological complete response, *PR* Progesterone receptor, *TN* Triple-negative

Breast tissue was processed for histopathological analysis according to standard anatomical pathology protocols in compliance with all College of American Pathologists standards. Tissues were fixed in 10% neutral buffered formalin and processed overnight in standard tissue processors. Slides were cut at 5 μm and stained on a Ventana automated staining system (Ventana Medical Systems, Tucson, AZ, USA). All pathological diagnoses were rendered by the breast pathology subspecialty service at University Hospitals Cleveland Medical Center (Cleveland, OH, USA). Pathological response to NAC was defined as an absence of invasive cancer in the breast surgical specimen (ypT0/is). Following the course of NAC, 36 patients achieved pCR in the breast as observed on the surgical specimen. Of these 36 patients who had a complete response in the breast (defined at T0 or Tis), 29 also were lymph node-negative at the time of surgery. Of the remaining seven patients with positive lymph nodes, all but one had only scant disease remaining in the lymph nodes following NAC (three were ypN1mi, three were ypN1a, and one was ypN2a). The remaining 81 patients had partial or no response to NAC in the breast (non-pCR).

Patients considered in this study had been scanned previously at different sites within the Cleveland University Hospitals network and on different scanner platforms with different magnets at both 1.5 T (MAGNETOM Avanto [Siemens Healthcare, Erlangen, Germany], MAGNETOM Espree [Siemens Healthcare], and Intera [Philips Medical Systems, Best, The Netherlands]) and 3 T (MAGNETOM Verio [Siemens Healthcare], Ingenia [Philips Medical Systems], and Ingenuity [Philips Medical Systems]). Axial fast low angle shot fat-saturated T1-weighted 3D scans were obtained before and after contrast agent injection. Pixel sizes ranged from 0.50 × 0.50 mm to 1.0 × 1.0 mm, with an average of 0.76 × 0.76 mm. Slice thickness ranged from 0.9 to 3 mm (average 1.3 mm).

An initial fat-saturated T1-weighted precontrast scan was first collected. Then 0.1 mmol/kg of either OptiMARK (Mallinckrodt, St. Louis, MO, USA) or MultiHance (Bracco Diagnostics, Monroe Township, NJ, USA) gadolinium-based contrast agent was injected intravenously. A first postcontrast scan was collected 2 minutes after contrast agent injection. Four subsequent postcontrast images were acquired at intervals of 90 seconds, resulting in five postcontrast images for each patient (*t* = 2, 3.5, 5, 6.5, and 8 minutes).

Two breast radiologists with 23 years (DP) and 3 years (ME) of experience reviewed and annotated lesion boundaries as the intratumoral region in consensus. A 2.5- to 5-mm (depending on pixel size) radius surrounding the tumor was defined as the peritumoral region. Lesion annotation and peritumoral mask generation are described in full in Additional file 1: Supplementary Methods.

### Feature extraction

All feature extraction was performed using software previously developed in the Center for Computational Imaging and Personalized Diagnostics, Case Western Reserve University, implemented on a MATLAB release 2016a platform (MathWorks, Natick, MA, USA). A total of 99 textural descriptors were computed voxel-wise across all slices for each phase, and location was analyzed. A list of textural features explored, along with descriptions and biological rationales, is provided in Table 2.

**Table 2:** Radiomic feature families extracted from the intratumoral and peritumoral regions

Feature group	Quantity	Description	Rationale
Laws energy measures	25	Response to 5-pixel × 5-pixel filter targeting combination of specific textural enhancement patterns in the *x* and *y* directions. Descriptors include all combinations of five 1D filters: level (L), edge (E), spot (S), wave (W), and ripple (R).	May possibly detect patterns of heterogeneous enhancement and abnormal structure; have previously been shown to enable quantification of TILs by lung CT [57].
Gabor features	48	Detection of edges through response to Gabor wavelet features. Each descriptor quantifies response to a given Gabor filter at a specific frequency (*f* = 0, 2, 4, 8, 16, or 32) and orientation (θ = 0 degrees, 22.5 degrees, 45 degrees, 67.5 degrees, 90 degrees, 112.5 degrees, 135 degrees, 167.5 degrees).	May possibly capture changes in tumor microarchitecture on account of glandular morphology or detect the presence of TILs [57]. TILs have been shown to be prognostic of better survival and NAC response [54].
Haralick features	13	Quantify heterogeneity and entropy of local intensity texture as represented by the gray-level co-occurrence matrix within a 5-pixel × 5-pixel window.	Regional changes in Haralick features following treatment have been shown to predict pCR in breast cancer [19].
Co-occurrence of Local Anisotropic Gradient Orientations (CoLlAGe) features	13	Apply Haralick metrics to dominant intensity gradient orientations within a 5-pixel × 5-pixel window, quantifying patterns of local gradient alignment [59, 60]. Some descriptors quantify homogeneity of gradient orientations (e.g., CoLlAGe information measure of correlation), whereas others compute their disorder (e.g., CoLlAGe entropy).	CoLlAGe entropy has previously been demonstrated to be effective in distinguishing breast cancer subtypes [59, 60].

*Abbreviations: CT* Computed tomography, *NAC* Neoadjuvant chemotherapy, *pCR* Pathological complete response, *TIL* Tumor-infiltrating lymphocyte

Physiologically based DCE-MRI pharmacokinetic (PK) parameters (rate of contrast agent uptake into tumor from blood [*K* ^Trans^], rate of contrast agent transport from tumor to blood [*K*_ep_], and tumor volume [V_e_]) were also computed from voxel intensities across each postcontrast phase using the Tofts model [35] for comparison against intratumoral and peritumoral radiomics.

First-order statistics (mean, median, SD, skewness, and kurtosis) of descriptor values were calculated for each location and phase-analyzed, resulting in 495 statistical features for each location and phase analyzed. A total of 1980 features for each scan were obtained, with 990 statistical features each extracted from the intratumoral and peritumoral regions. Feature values were normalized between −1 and 1. 

All quantitative data analysis was implemented using MATLAB release 2016a software unless otherwise stated.

### Experiment 1: pCR prediction among all-comers

Groups of 24 pCR and 54 non-pCR patients were randomly sorted into a 78-patient training set. The remaining 39 patients, including 12 pCR and 27 non-pCR, were held out as the independent testing set. Training and testing set information is included in Table 3.

**Table 3:** Breakdown of dataset by experiment

	Experiment 1: training	Experiment 1: testing	Experiment 2: HR^+^, HER2^−^	Experiment 3: TN/HER2^+^
Number of patients	78	39	70	47
Type of pathological response				
pCR	24	12	10	26
Non-pCR	54	27	60	21
Receptor group				
HR^+^, HER2^−^	46	24	70	0
TN/HER2^+^	32	15	0	47
Scanner strength				
1.5 T	63	27	54	36
3 T	15	12	16	11

*Abbreviations: HER2* Human epidermal growth factor receptor 2, *HR* Hormone receptor, *pCR* Pathological complete response, *TN* Triple-negative

#### Feature discovery

Minimum redundancy, maximum relevancy (mRMR) feature selection [36] was implemented to select the top 10 pCR-associated features across 200 iterations of threefold cross-validation within the training cohort. mRMR was used to identify a set of features that maximally distinguished two classes (pCR and non-pCR) while minimizing intrafeature correlation. Features were ranked by frequency of selection, and the bottom 90% of features were eliminated. Feature selection was repeated within the reduced feature set, and features were again ranked by selection frequency. Redundant overlapping features were eliminated from this list to yield a final set of top pCR-associated radiomic features. The number of features selected was capped at ten to prevent overfitting due to the “curse of dimensionality” [37] arising from an overabundance of features relative to sample size. mRMR was performed using MATLAB software with the Feature Selection Toolbox for C (FEAST; Czech Academy of Sciences, Prague, Czech Republic) [38].

This top radiomic feature set was analyzed using box-and-whisker plots and qualitative feature maps comparing feature expression between representative pCR and non-pCR tumors. Box plots for the top feature identified were compared against the performance of PK parameters that are physiological measures of tumor contrast uptake. Hematoxylin and eosin staining of pretreatment diagnostic core biopsy specimens for representative patients was also qualitatively examined by a breast pathologist (HG) with 10 years of experience to explore and potentially identify a possible physiological basis for the top-expressing radiomic features.

#### Validation

Two distinct strategies were used to validate our intratumoral and peritumoral radiomic features set. First, to validate the importance of a combined intratumoral and peritumoral radiomic approach in identifying pCR in a biologically heterogeneous dataset, consensus clustering [39] of the top ten intratumoral and peritumoral radiomic features was performed within the training group using the ConsensusClusterPlus package [40] in R [41]. One thousand iterations of hierarchical consensus clustering (*k* = 2) by Pearson distance [42] were performed with 80% random patient resampling between runs. Clustering results were visualized in a consensus cluster heatmap where shading indicated the consensus between patients or the frequency at which a pair of patients was clustered together. Clustering results were compared against pCR ground truth to assess the ability of top features to identify responders without a priori knowledge of patient biology or outcome. Feature discovery and consensus clustering were repeated in the subsets of intratumoral and peritumoral features only and compared against a combined clustering of intratumoral and peritumoral radiomic features. Consensus clustering was also performed and compared using all 15 PK statistical features from the entire dataset.

Next, we assessed the ability of an intratumoral and peritumoral radiomic feature set to predict pCR among new patients. The ability of top features to predict pCR was validated using five different machine learning classifiers to verify that successful pCR prediction was driven by our features as opposed to the choice of classifier. The following classifiers [43] were explored: linear discriminant analysis (LDA), diagonal linear discriminant analysis (DLDA), quadratic discriminant analysis, naive Bayes, and support vector machine. Within the training group, classifiers were iteratively trained and applied across 50 iterations of threefold cross-validation to first assess pCR prediction within the training set. Classifiers were then trained on the training set and tested by prediction of response within the independent testing set. Training and classification of each classifier was repeated ten times to sequentially include the top ten radiomic features. Performance was assessed by AUC, accuracy, sensitivity (ability to correctly identify patients who achieved pCR), and specificity (ability to correctly identify patients who did not achieve pCR).

### Experiment 2: pCR prediction with separation by receptor type

Table 3 lists receptor status groups evaluated in experiment 2. The subset of 70 patients (10 pCR, 60 non-pCR) who were estrogen receptor (ER)- or progesterone receptor (PR)-positive, as well as HER2^−^, were grouped into a hormone receptor (HR)-positive, HER2^−^ (HR^+^, HER2^−^) cohort. The remaining 47 patients included 19 triple-negative (TN) and 28 HER2^+^ tumors, and these patients were analyzed as a combined 47-patient TN/HER2^+^ cohort (26 pCR and 21 non-pCR). Owing to a low response rate within the HR^+^, HER2^−^ group and fewer total samples within the TN/HER2^+^ group, neither was divided into training and testing groups. Instead, for experiment 2 alone, threefold cross-validation was used for classifier training and testing.

#### Feature discovery

Feature discovery was repeated among the entire subset of patients with HR^+^, HER2^−^ breast cancer. Because only 10 of the 70 HR^+^, HER2^−^ patients experienced pCR, non-pCR tumors were randomly downsampled to a group of 20 for each iteration of mRMR cross-validation to reduce selection of feature sets biased toward non-pCR samples. Feature discovery was performed within the group of TN/HER2^+^ patients without downsampling. Features were evaluated using box plots and qualitative feature maps.

#### Validation

Classifiers were trained in 50 iterations of threefold cross-validation using the top 1 through 10 features identified within each receptor status group. pCR prediction was assessed by mean AUC, accuracy, sensitivity, and specificity across cross-validation repetitions.

## Results

### Experiment 1: pCR prediction among all-comers

#### Feature discovery

The top ten radiomic feature set obtained during feature discovery within the training set are listed in Table 3. CoLlAGe features are most frequently represented, occupying six of the top features. The remaining four members are Laws features. Intratumoral features and features extracted from the initial postcontrast phase are both more prevalent within the top feature set, each comprising six top features. Additionally, the top two features, CoLlAGe information measure of correlation 1 and Laws spot-ripple, were both expressed intratumorally during the first postcontrast phase.

pCR was most frequently characterized by increased intratumoral kurtosis of CoLlAGe information measure of correlation 1 during the initial enhancement phase, a feature quantifying homogeneity of intensity gradient orientations. In Fig. 3a, increased pCR expression of CoLlAGe information measure of correlation 1 within the intratumoral region during the initial postcontrast phase is shown in representative feature maps. The histology of representative patients revealed a high presence of tumor-infiltrating lymphocytes (TILs) in pCR, as well as sclerosis and necrosis in non-pCR biopsy samples (Fig. 3d). Box plots show elevated expression among patients who experienced pCR as compared with non-pCR in both the training and testing sets (Additional file 2). Features 2 and 3—skewness of intratumoral initial Laws spot-ripple and SD of peritumoral peak Laws level-spot—showed that pCR and non-pCR differed in patterns of enhancement texture at both phases and locations. The fourth top feature—kurtosis of CoLlAGe sum entropy—was expressed more strongly within the peritumoral region of nonresponders during the initial enhancement phase.

#### Validation

**Fig. 2:**
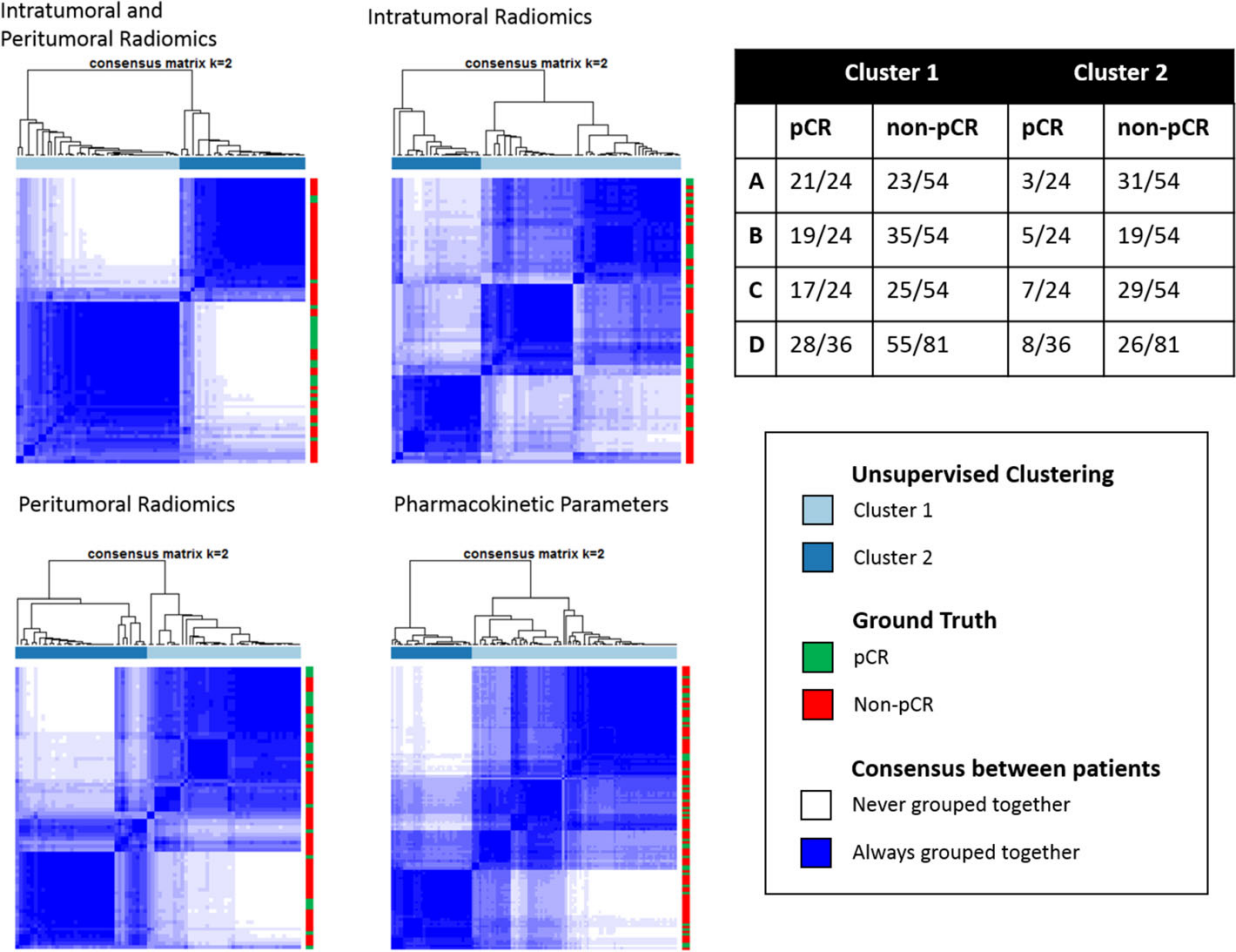
Consensus clustering using combined peritumoral and intratumoral radiomics, intratumoral radiomics, peritumoral radiomics, and pharmacokinetic parameter feature sets. Combination of intratumoral and peritumoral features yielded clusters with the best consensus and correlation to pathological complete response (pCR) status

In Fig. 2a, the combined intratumoral and peritumoral feature set yields distinct pCR-associated clusters, with 88% pCR and 57% non-pCR clustering accuracy. In Fig. 2b, consensus clustering using a set of the top ten intratumoral features produced groups with weak consensus and correlation with pCR, with cluster 1 receiving a majority of both pCR (79%) and non-pCR (65%) tumors. Clustering performed using only peritumoral features was less accurate than the combined peritumoral and intratumoral clustering (71% pCR and 54% non-pCR clustering accuracy) and had noticeably weaker consensus within clusters, as shown in Fig. 2c. PK parameters produced clusters with poor consensus and approximately proportional distribution of response groups (cluster 1 78% pCR, 69% non-pCR), as shown in Fig. 2d. Additionally, box plots of mean *K* ^Trans^, *K*_ep_, and V_e_ (Additional file 2) showed no difference between pCR and non-pCR tumors for any of the experimental groups examined in this study.

Optimal classifier performance within the training set was achieved using a DLDA trained with 10 features across 50 threefold cross-validation iterations, yielding an average AUC of 0.78 ± 0.03 and accuracy of 0.76 ± 0.03. Prediction accuracy did not differ between patients using 1.5-T and 3-T MRI scanners. Within the testing set, a DLDA classifier trained using only the top four features produced the strongest classification results. (AUC = 0.74, accuracy = 0.67). Of the 13 misclassified tumors, 7 were HR^+^, HER2^−^ nonresponders misidentified as pCR. The remaining six tumors were TN/HER2^+^ (66% true responders and 33% true nonresponders). The classifier’s accuracy within the testing set was greater for HR^+^, HER2^−^ tumors than for TN/HER2^+^ tumors (0.71 vs. 0.60). The 6 misclassified TN/HER2^+^ cases were comprised of 2 of the 4 TN tumors (accuracy = 0.50) and 4 of the 11 HER2^+^ tumors (accuracy = 0.64) within the testing set. The addition of subsequent features resulted in a decrease of both AUC and accuracy within the testing set. The top four radiomic features were successful in predicting pCR within the testing set regardless of classifier selection (Additional file 3: Tables S1–S5). Seven of thirteen misclassifications were imaged with a 3-T scanner.

### Experiment 2: pCR prediction with separation by receptor type

#### Feature discovery

**Fig. 3:**
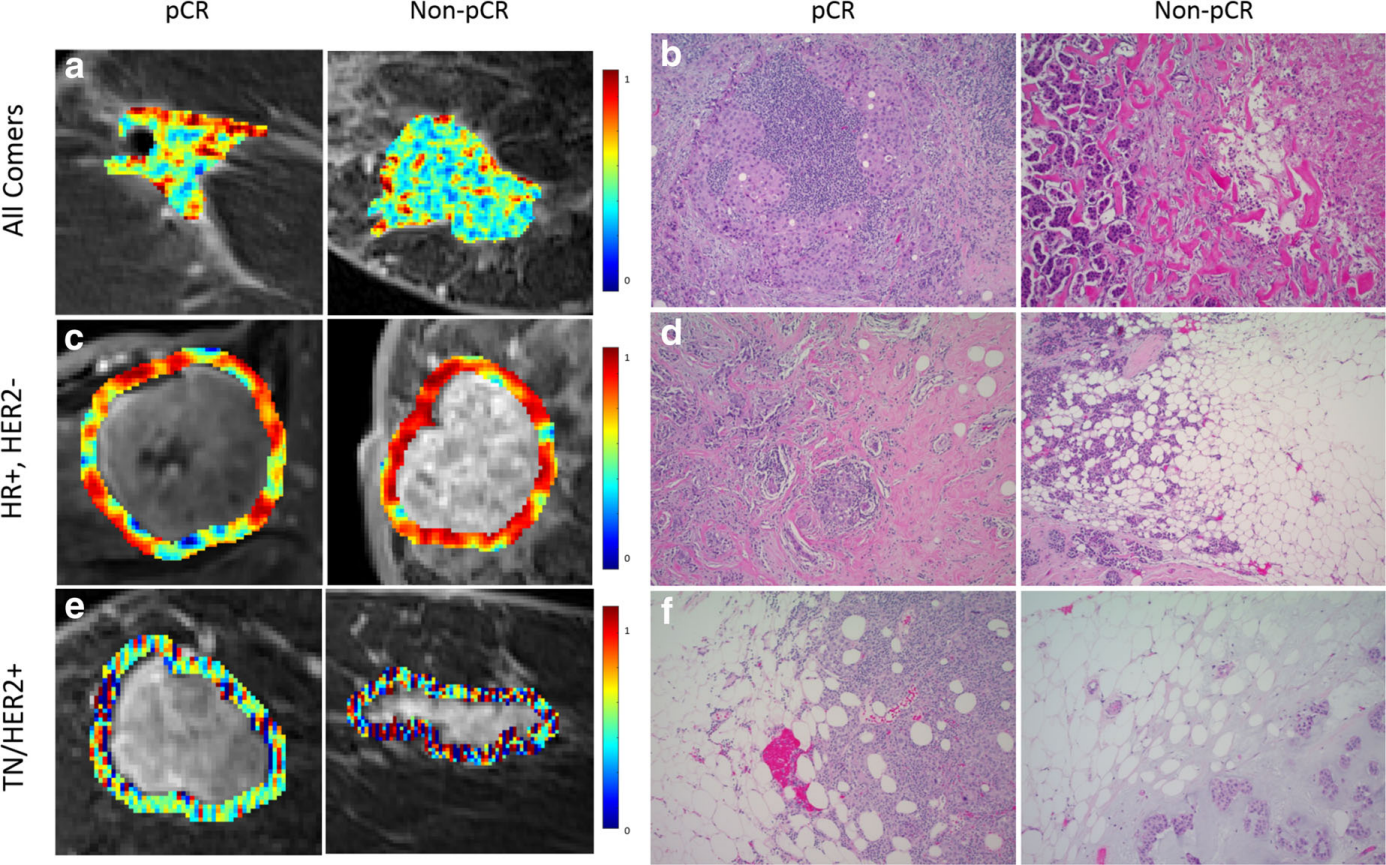
**a**, **c**, **e** Feature expression maps for top radiomic features. **b**, **d**, **f** Corresponding hematoxylin and eosin-stained images at × 100 original magnification taken from the original diagnostic core biopsy specimen before neoadjuvant chemotherapy. *All-comers:* **a** Co-occurrence of Local Anisotropic Gradient Orientations (CoLlAGe) information measure of correlation 1 is elevated in pathological complete response (pCR) tumors intratumorally during the initial postcontrast phase. **b** For a patient who experienced pCR, the corresponding histology shows a high percentage of stromal tumor-infiltrating lymphocytes (TILs) present relatively uniformly within the invasive carcinoma. The histopathological image from the non-pCR patient on the *right* shows a heterogeneous mix of tumor cells, necrosis, and sclerosis. *HR* ^*+*^ */HER2* ^*−*^ *:* **c** Peritumoral initial CoLlAGe entropy is increased among HR^+^, HER2^−^ nonresponders. **d** Corresponding histology for peritumoral regions of HR^+^/HER2^−^ patients with and without a pCR. The image on the *left* shows a brisk lymphocytic response at the periphery of the tumor. The image on the *right* from the non-pCR patient shows tumor cells infiltrating the adipose tissue at the periphery of the lesion without a significant stromal response. *TN/HER2* ^*+*^ *:* **e** Peritumoral peak Laws level-ripple is elevated in non-pCR tumors. **f** Peritumoral regions from patients with TN breast cancer with and without a pCR. Once again, there is a brisk lymphocytic response in the peritumoral region and numerous stromal TILs within the tumor on the *left*. The image of the non-pCR on the *right* is from a patient with TN breast cancer with a matrix-producing metaplastic carcinoma. The tumor cells with associated chondroid matrix are dissecting through the adipose tissue at the periphery of the lesion. *HER2* Human epidermal growth factor receptor 2, *HR* Hormone receptor, *TN* Triple-negative

Half of the top ten HR^+^, HER2^−^ feature set obtained from feature discovery comprised CoLlAGe features. Laws, Gabor, and Haralick features were all represented within the top feature set as well. Six features were extracted from the peritumoral region and six from the initial post-contrast enhancement phase. The top three most discriminating features were all CoLlAGe entropy-associated descriptors expressed within the peritumoral region during the initial enhancement phase: kurtosis of entropy, skewness of difference entropy, and skewness of sum entropy. Top CoLlAGe feature expression indicates a more disordered peritumoral DCE-MRI phenotype among non-pCR HR^+^, HER2^−^ tumors, particularly during the initial phase of post-contrast enhancement. Figure 3b shows qualitative feature maps of representative initial peritumoral CoLlAGe entropy for pCR and non-pCR breast cancers. In Fig. 3e, histology of a low CoLlAGe entropy-expressing pCR tumor shows brisk lymphocytic response at the tumor margins. Conversely, a non-pCR sample with high peritumoral entropy had significant tumor infiltration into surrounding adipose tissue. Additionally, intratumoral expression of low-frequency Gabor features was elevated during both initial and peak enhancement phases (features 6 and 8).

TN/HER2^+^ responder tumors were predominantly defined by Laws and CoLlAGe features, each comprising four of the ten top radiomic features. All top Laws features were computed peritumorally, with textural patterns of inconsistent enhancement such as waves, edges, and spots distinguishing pCR and non-pCR in both initial (spot-level, level-wave) and peak (level-ripple, edge-spot) enhancement phases. The top TN/HER2^+ ^feature was the expression of Laws level-ripple during peak enhancement phase, shown qualitatively in Fig. 3c. Significant TIL presence was observed in a pCR sample with low Laws rippling, whereas high expression was found to correspond with a tumor with invasion of chondroid matrix into surrounding adipose tissue. Note that increased rippling in the peritumoral region of non-pCR tumors results in increased extreme positive and negative filter response values, whereas the more homogeneous pCR peritumoral appearance yields more consistent midrange values. CoLlAGe features quantifying homogeneity of orientation gradients—most notably initial intratumoral information measure of correlation 1, the top feature discovered in experiment 1—comprised features 2, 3, and 4. CoLlAGe gradient orientation features were discriminative both intratumorally during initial phase (features 2 and 4) and peritumorally during peak enhancement (feature 3). Two low-frequency Gabor features were also discriminative within the intratumoral region.

The top ten features identified within the all-comers, HR^+^ HER2^−^, and TN/HER2^+^ groups are included in Table 4. Note that the two highest-ranked features among all-comers were located within the intratumoral region; however, peritumoral features were ranked highest when feature discovery was performed within receptor type-specific groups.

**Table 4:** Top ten radiomic features identified in each experiment

Rank	Feature family	Descriptor	Location	Phase	Statistic
Experiment 1					
All-comers					
1	CoLlAGe	Information measure of correlation 1	Intratumoral	Initial	Kurtosis
2	Laws	Spot-ripple	Intratumoral	Initial	Skewness
3	Laws	Level-spot	Peritumoral	Peak	SD
4	CoLlAGe	Entropy	Peritumoral	Initial	Kurtosis
5	CoLlAGe	Sum average	Intratumoral	Peak	SD
6	Laws	Edge-wave	Peritumoral	Initial	Mean
7	CoLlAGe	Energy	Intratumoral	Initial	Median
8	Laws	Wave-spot	Intratumoral	Initial	Skewness
9	CoLlAGe	Inverse difference moment	Peritumoral	Peak	Kurtosis
10	CoLlAGe	Correlation	Intratumoral	Peak	Kurtosis
Experiment 2					
HR^+^, HER2^−^					
1	CoLlAGe	Entropy	Peritumoral	Initial	Kurtosis
2	CoLlAGe	Difference entropy	Peritumoral	Initial	Skewness
3	CoLlAGe	Sum entropy	Peritumoral	Initial	Skewness
4	Laws	Level-ripple	Intratumoral	Peak	Kurtosis
5	CoLlAGe	Inertia	Intratumoral	Peak	Skewness
6	Gabor	*f* = 0, θ = 22.5 degrees	Intratumoral	Initial	Median
7	Laws	Edge-edge	Peritumoral	Initial	Skewness
8	Gabor	*f* = 2, θ = 22.5 degrees	Intratumoral	Initial	Kurtosis
9	CoLlAGe	Inverse difference moment	Peritumoral	Peak	Skewness
10	Haralick	Correlation	Peritumoral	Peak	SD
TN/HER2^+^					
1	Laws	Level-ripple	Peritumoral	Peak	Mean
2	CoLlAGe	Information measure of correlation 1	Intratumoral	Initial	Skewness
3	CoLlAGe	Information measure of correlation 2	Peritumoral	Peak	Mean
4	CoLlAGe	Energy	Intratumoral	Initial	Median
5	Laws	Spot-level	Peritumoral	Initial	Median
6	Gabor	*f* = 2, θ = 135 degrees	Intratumoral	Peak	Kurtosis
7	Laws	Level-wave	Peritumoral	Initial	Median
8	Laws	Edge-spot	Peritumoral	Peak	Skewness
9	CoLlAGe	Difference variance	Peritumoral	Peak	Kurtosis
10	Gabor	*f* = 2, θ = 22.5 degrees	Intratumoral	Initial	Skewness

*Abbreviations: CoLlAGe* Co-occurrence of Local Anisotropic Gradient Orientations, *HER2* Human epidermal growth factor receptor 2, *HR* Hormone receptor, *TN* Triple-negative

#### Validation

Subtype-specific prediction vastly improved the ability to predict pCR. Optimal response prediction was achieved with a ten-feature DLDA classifier, predicting pCR among HR^+^, HER2^−^ tumors with an AUC of 0.83 ± 0.025 and an accuracy of 0.79 ± 0.033. Classification sensitivity and specificity were 0.65 ± 0.076 and 0.82 ± 0.038, respectively. The ability of intratumoral and peritumoral radiomic features to predict pCR was shown to be robust across multiple classifiers (Additional file 3: Tables S1–S5).

A naive Bayes classifier trained with eight features was able to predict pCR with an average AUC of 0.93 ± 0.018 and accuracy of 0.84 ± 0.030. Sensitivity was 0.87 ± 0.030, and specificity was 0.81 ± 0.047. The accuracy of pCR prediction among TN tumors was 0.79 ± 0.041, whereas HER2^+^ accuracy was 0.88 ± 0.041. The ability to predict pCR was consistent across all tested classifiers (Additional file 3: Tables S1–S5), including DLDA (AUC = 0.89 ± 0.027, accuracy = 0.83 ± 0.030).

## Discussion

In this study, we investigated the ability of radiomic textural features extracted from the intratumoral and peritumoral regions of pretreatment DCE-MRI to predict pCR to NAC. This approach was able to accurately distinguish between pCR and non-pCR in a highly heterogeneous cohort of pretreatment DCE-MRI scans. Most prior breast cancer radiomic approaches have been focused solely within the extent of the tumor itself [12–21, 24, 25] or the bulk parenchyma [22, 26–28]. The immediate surrounding tumor environment has remained relatively unexplored and may offer unique, orthogonal radiomic signatures of response prior to administration of NAC that enable enhanced pCR prediction.

In the feature discovery portion of this study, we attempted to define radiomic profiles of NAC responders. We found that CoLlAGe features measuring the consistency of local dominant gradient orientation differentiated pCR both intra- and peritumorally across all subtypes. Tumors that achieved pCR were characterized by increased expression of gradient homogeneity features within the tumor and decreased expression of gradient entropy features at the perimeter. Pretreatment diagnostic biopsy core samples for patients with representative radiomic expression (Fig. 3) revealed densely packed stromal TILs within the tumor that achieved pCR, likely contributing to a greater order of intratumoral intensity gradients as detected by CoLlAGe. This result is consistent with previous findings associating high TIL presence with more homogeneous DCE-MRI enhancement within the context of TN breast cancers [44]. The non-pCR tumor was found to contain intermingled tumor cells, necrosis, and sclerosis, likely contributing to the heterogeneity of the imaging appearance detected by CoLlAGe. These features might also be detecting enhancement heterogeneity due to increased chaotic angiogenesis within and surrounding tumors that do not achieve pCR; however, vascular marker staining of patient biopsies was not available to verify this hypothesis. Laws features capturing patterns of inconsistent enhancement such as speckling and rippling within the intratumoral and peritumoral regions were also found to discriminate between pCR and non-pCR.

We demonstrated that a combined intratumoral and peritumoral radiomic approach yielded clusters with high consensus and strong correlation with pCR ground truth in an unsupervised consensus clustering, outperforming radiomic analysis confined to a single region and physiologically based PK parameters. A DLDA classifier trained using the top four features identified during feature discovery predicted pCR with an AUC of 0.74 among the independent testing cohort. These features were successful in predicting pCR regardless of classifier chosen, suggesting that the features identified are robust predictors of response. These findings suggest that a combination of intratumoral and peritumoral cues may provide a more accurate and more comprehensive radiomic profile for characterization of breast cancers based on MRI.

Considering these results, we sought to determine whether the ability to predict response would improve with receptor status-specific feature sets and classifiers. In experiment 2, separating tumors by receptor status and repeating feature discovery improved the ability of an intratumoral and peritumoral radiomic classifier to predict pCR. Among HR^+^, HER2^−^ tumors, a group strongly correlated with the luminal A molecular subtype [45], we could predict pCR with an AUC = 0.83 ± 0.025. Owing to insufficient sample sizes of both TN and HER2^+^ patients, we grouped these patients into a combined TN/HER2^+^ cohort. Relative to HR^+^, HER2^−^ breast cancers, these subtypes more frequently achieve pCR [46], and their response to NAC is more accurately detected on the basis of post-NAC MRI [47]. We demonstrated that separation of TN/HER2^+^ enabled more accurate prediction of pCR (AUC = 0.93 ± 0.018) than in the HR^+^, HER2^−^ cohort, a surprising reversal of the receptor status group accuracies reported in experiment 1.

Not only was pCR predicted more accurately using a receptor type-specific classifier but different biological groups also were found to possess unique radiomic profiles of response. HR^+^, HER2^−^ breast cancers were defined strongly by CoLlAGe features associated with entropy, whose expression was elevated in the peritumoral region of non-pCR tumors. An HR^+^, HER2^−^ patient with pCR and low peritumoral expression of CoLlAGe entropy features was observed to have a brisk lymphocytic response at the tumor’s periphery. The non-pCR sample possessed significant infiltration of tumor cells into surrounding adipose tissue, potentially contributing to its high CoLlAGe-detected gradient entropy within the peritumoral region. These features could be detecting increasingly disordered enhancement gradients among nonresponders because of increased peritumoral vascularity and vascular invasion, an established marker of poor prognosis and risk of recurrence in HR^+^, HER2^−^ breast cancers [48, 49]. It is possible that peritumoral gradient orientation entropy during the initial enhancement phase was dominant within the top HR^+^, HER2^−^ feature set because HR^+^, HER2^−^ tumors enhance more gradually than other subtypes [50, 51], making peritumoral vasculature abnormalities of nonresponders more apparent at the first postcontrast phase as compared with those who achieve pCR. Low-frequency wavelet features (Gabor) were also observed intratumorally, which have previously been found to detect architectural disorder of mammary glands on breast mammograms [52, 53].

In the TN/HER2^+^ cohort, Laws feature expression within the peritumoral region was found to be valuable in predicting pCR. These features might be detecting textural patterns caused by surrounding stromal response or the presence of TILs at the tumor periphery, a predictor of pCR in both HER2^+^ [54, 55] and TN [54, 56] breast cancers, because Laws features have previously been shown to enable quantification of TILs in other imaging domains [57]. Examination of pretreatment biopsy samples substantiated this possibility, revealing brisk lymphocytic response and significant presence of TILs in a patient with pCR and low rippling in the peritumoral region as detected by Laws features. Conversely, the non-pCR patient observed to have the highest prevalence of rippling in the peritumoral region was discovered to have a matrix-producing metaplastic carcinoma with chondroid matrix infiltrating surrounding adipose tissue. Although not all TN/HER2^+^ non-pCR patients possess this phenotype, this finding does suggest that infiltration into the adipose tissue may manifest as a rippled pattern detectable using Laws features. CoLlAGe features within both the intratumoral and peritumoral regions, as well as low-frequency intratumoral Gabor features, also helped define the TN/HER2^+^ responder phenotype. TN and HER2^+^ breast cancers were also analyzed separately (detailed in Additional file 4: TN-specific and HER2^+^-specific response prediction) to compare the identified radiomic response features for the individual molecular subgroups with those identified when the TN and HER2^+^ cases were combined. We found that radiomic response features for the TN group to comprise Haralick, CoLlAGe, and Laws features from both the peritumoral and intratumoral regions, whereas the HER2^+^ response signature was composed almost entirely of Laws features from the peritumoral region during the peak enhancement phase (Additional file 4: Table S6). Response was predicted with average AUCs of 0.82 for TN and 0.80 for HER2^+^, as compared with an AUC of 0.80 for combined TN/HER2^+^ when using a comparable number of features and classifiers (Additional file 4: Table S7).

The work presented in this study did have its limitations, however. Although distinct training and testing cohorts were used in experiment 1, we were unable to implement testing sets within the HR^+^, HER2^−^, and TN/HER2^+^ subgroups, owing to insufficient HR^+^, HER2^−^ responders and total TN/HER2^+^ patients. AUCs reported for receptor status group-specific pCR prediction in experiment 2 were obtained by averaging cross-validation results within the same pool of patients used for the feature discovery phase (also performed using threefold cross-validation). As in experiment 1, these AUCs could decrease when evaluated in an independent testing cohort. However, receptor status group-specific AUCs still improved as compared with results of cross-validation within a training cohort of all-comers (AUC = 0.78 ± 0.03), suggesting that receptor status group-specific analysis would likely also enable stronger pCR prediction in an independent testing set. It is also possible that the elevated AUC within the TN/HER2^+^ cohort could be partially due to a reduced sample size (*n* = 47) compared with the HR^+^, HER2^−^ (*n* = 70) and all-comer training cohorts (*n* = 78); however, this result is consistent with previous findings that MRI has greater value in response prediction for TN/HER2^+^ patients [47]. Our dataset also included diversity of treatment regimens within the all-comer and TN/HER2^+^ groups, which might impact the likelihood of achieving pCR. In addition to the variability in prescribed treatment, the TN/HER2^+^ group featured two distinct receptor status groups. TN and HER2^+^ were combined into a single group, both on the basis of their correspondence to a nonluminal phenotype [45] and owing to the limited sample size. pCR was predicted in the TN/HER2^+^ group with the highest AUC of any of the patient groups analyzed, suggesting that these cancers seem to possess shared radiomic response signatures. However, these subtypes nonetheless represent distinct biologies and receive different treatment regimens. Grouping these tumors might possibly cause radiomic features that capture response signatures unique to HER2^+^ or TN breast cancers to be overlooked. For instance, radiomic features that detect HER2 enrichment, a predictor of improved response to HER2-targeted therapy [58], might strongly predict pCR in HER2^+^ tumors, but they would likely have little predictive value for TN breast cancer. As a result, these features might be missed during feature discovery in a combined TN/HER2^+^ dataset. Indeed, feature discovery within separated TN and HER2^+^ groups produced differing response signatures (Additional file 4: Table S6). In future work, we intend to explore TN-specific and HER2^+^-specific radiomic response signatures and their relationship with unique subtype biology with larger sample sizes. Additionally, our dataset is highly diverse with respect to the DCE-MRI protocol, with a multitude of scanners and heterogeneous magnet strengths. Although the rate of misclassification within the training set did not vary with magnet strength; 3-T scans were disproportionately misclassified in the testing set. Further investigation is required to determine the role that magnet strength may play in intratumoral and peritumoral radiomics. Owing to variance in voxel dimensions among scans, the dimensions of the peritumoral region occupies anywhere between a 2.5- to 5.0-cm radius from the tumor. Further, the size of the peritumoral region varies widely on the basis of biological and clinical factors; therefore, approximating the peritumoral radius as a consistent distance from the tumor is an imperfect means of obtaining the peritumoral region. Analysis within recommended negative surgical margins was deemed a suitable stand-in for true peritumoral region borders that allowed quick sampling with minimum required clinical input. Future exploration of radiomic analysis within radiologist-annotated peritumoral regions may further improve the results presented in this study.

This work represents a preliminary success for the pretreatment prediction of pCR using intratumoral and peritumoral radiomics, which could potentially help guide personalized treatment of locally advanced breast cancers and reduce windows of ineffective treatment. Much work remains to be done, however, before its realization as a clinical tool. Although we were able to successfully demonstrate our ability to predict pCR among all-comers in experiment 1 with an independent holdout testing set, both these findings and the improved results of subtype-specific classifiers must next be validated within a larger independent dataset from possibly multiple external sites. Previous work [13] has suggested that radiomic features might be minimally affected by factors such as MRI magnetic strength. However, the Agner et al. study [13] was a limited study, and future work will entail a more detailed interrogation of the impact of scan parameters within the context of radiomic NAC response signatures. Further investigation may reveal the need for new standardization approaches to mitigate the effects of scan parameter heterogeneity. Additionally, greater intuition regarding the underlying biology captured by radiomic features will be a critical factor in their widespread adoption as clinical decision support tools. In this work, we conducted a qualitative assessment of representative patient histology in an attempt to unravel the morphometric and biological basis for the most predictive radiomic features presented. Future directions could include correlating radiomic features with both features from corresponding digital pathology surgical tissue sections and genomic measurements to more comprehensively characterize and identify the morphological and molecular basis of the most NAC-predictive radiomic features. Translation of this work will additionally require development of a platform with an intuitive user interface so that radiomic analysis could be easily implemented by doctors and integrated into current clinical workflows.

## Conclusions

Patients with breast cancer who achieve pCR with NAC have a significantly improved prognosis and disease-free survival; however, only 10–50% of NAC recipients will achieve a full response [6]. There is a lack of imaging markers that enable noninvasive pretreatment prediction of pCR. In this study, we employed a novel combined intratumoral and peritumoral radiomic approach for pCR prediction, combining textural features extracted from a tumor and its immediate surrounding environment based on routine clinical DCE-MRI imaging. The efficacy of the approach was demonstrated in a relatively large and diverse imaging dataset. Specifically, we showed that peritumoral radiomics contribute to successful prediction of pCR from pretreatment imaging. Further, we identified molecular subtype-specific radiomic profiles of response and found that radiomic features most predictive of response appear to vary as a function of the molecular subtype of the tumor. These findings potentially hold significant clinical applications because they could help enable a clinical platform for the pretreatment prediction of pCR that would allow identification of patients most likely to achieve a response.

## Additional file

Additional file 1: supplementary methods. (DOCX 12.3 kb)
Additional file 2: Comparison of top radiomic and pharmacokinetic DCE-MRI parameters. Left: Box plots of top radiomic features among experimental groups. All-comers: kurtosis of intratumoral initial CoLlAGe information measure of correlation 1. HR^+^, HER2^−^: kurtosis of initial peritumoral CoLlAGe entropy. TN/HER2^+^: median of peritumoral peak Laws Level-Ripple. Right: Box plots of pharmacokinetic parameters (Ktrans, Kep, Ve) do not separate pCR and non-pCR among entire dataset, nor within HR^+^, HER2^−^ and TN/HER2^+^ groups. (PNG 186 kb)
Additional file 3: Table S1. Linear discriminant analysis (LDA) results for all experiments. **Table S2.** Diagonal linear discriminant analysis (DLDA) results for all experiments. **Table S3.** Support vector machine (SVM) results for all experiments. **Table S4.** Naïve Bayes results for all experiments. **Table S5.** Quadratic discriminant analysis (QDA) results for all experiments. (DOCX 35.9 kb)
Additional file 4: TN-specific and HER2^+^-specific response prediction. **Table S6.** Feature Discovery: HER2^+^ and TN tumors. Additional feature discovery experiments were performed for the subsets of HER2^+^ patients only (n=28) and TN patients only (n=19). Due to the limited sample size, only 5 top radiomic features were identified for each subtype. **Table S7.** Validation: HER2^+^ and TN tumors. The HER2^+^ and TN-specific signatures were evaluated by three-fold cross-validation of a DLDA classifier within the corresponding subset of patients and assessed by average AUC. Also shown are the results of using the top 5 features identified in the combined TN/HER2^+^ group for a DLDA classifier in a cross-validation setting within the combined TN/HER2^+^ group (n=47), HER2^+^ only group (n=28), and TN only group (n=19). (DOCX 17.2 kb)

## Abbreviations

CoLlAGe: Co-occurrence of Local Anisotropic Gradient Orientations
CT: Computed tomography
DCE-MRI: Dynamic contrast-enhanced magnetic resonance imaging
DLDA: Diagonal linear discriminant analysis
ER: Estrogen receptor
HER2: Human epidermal growth factor receptor 2
HIPAA: Health Insurance Portability and Accountability Act of 1996
HR: Hormone receptor
LDA: Linear discriminant analysis
mRMR: Minimum redundancy, maximum relevancy
NAC: Neoadjuvant chemotherapy
pCR: Pathological complete response
PK: Pharmacokinetic
PR: Progesterone receptor
TIL: Tumor-infiltrating lymphocyte
TN: Triple-negative
