# Erratum to: Intratumoral and peritumoral radiomics for the pretreatment prediction of pathological complete response to neoadjuvant chemotherapy based on breast DCE-MRI

**DOI:** 10.1186/s13058-017-0862-1

**Published:** 2017-07-10

**Authors:** Nathaniel M. Braman, Maryam Etesami, Prateek Prasanna, Christina Dubchuk, Hannah Gilmore, Pallavi Tiwari, Donna Plecha, Anant Madabhushi

**Affiliations:** 1 Department of Biomedical Engineering, Case Western Reserve University, Cleveland, OH 44106 USA; 2 University Hospitals Case Medical Center, Cleveland, OH 44106 USA

## Erratum

This article [1] has been updated to correct the spelling of one of the author’s names. Donna Plecha’s name was incorrectly spelt as Pletcha when this article originally published.
