# Supplementary material for: Intratumoral and peritumoral radiomics for the pretreatment prediction of pathological complete response to neoadjuvant chemotherapy based on breast DCE-MRI
Source: Breast Cancer Res. 2017 May 18;19:57. doi: 10.1186/s13058-017-0846-1 (PMC5437672; doi:10.1186/s13058-017-0846-1)
Supplement: Additional file 1: — supplementary methods. (DOCX 12.3 kb) [file 13058_2017_846_MOESM1_ESM.docx]

Additional File 1 – Supplementary Methods

Segmentation of Intratumoral and Peritumoral Regions

Two breast radiologists with 23 (D.M.P) and 3 years of experience (M.E.) reviewed and annotated patient scans using SECTRA IDS7/dx workstation in consensus. Images were manually delineated by M.E. and then individually reviewed by D.M.P. Cases of disagreement were resolved by consensus. Tumor boundaries were manually delineated on the initial enhancement phase across three adjacent representative slices with largest tumor area. Phase of peak enhancement was determined by the radiologist qualitatively via visual comparison.

A mask of the area within radiologist-annotated tumor boundaries was analyzed as the intratumoral region. From the intratumoral annotation, a second mask containing the peritumoral region was generated. The intratumoral mask was dilated at a radius of 5 pixels, equivalent to a 2.5 to 5 mm (depending on pixel size) region surrounding the tumor. This radius was corresponds with the 2 mm of surrounding negative tumor margin recommended for removal in breast conserving surgery followed by radiation therapy in the treatment of ductal carcinoma in situ by Society of Surgical Oncology–American Society for Radiation Oncology–American Society of Clinical Oncology Consensus Guidelines to prevent recurrence [1]. A mask of the breast wall and surrounding air was obtained through thresholding and a series of morphological operations (closing, erosion, and dilation). Pixels included in the original intratumoral mask or breast wall mask were eliminated from the dilated mask, leaving a mask containing a ring of breast tissue analyzed as the peritumoral region.

References

1. Morrow M, Zee KJV, Solin LJ, Houssami N, Chavez-MacGregor M, Harris JR, et al. Society of Surgical Oncology–American Society for Radiation Oncology–American Society of Clinical Oncology Consensus Guideline on Margins for Breast-Conserving Surgery With Whole-Breast Irradiation in Ductal Carcinoma In Situ. J. Clin. Oncol. 2016;JCO683573.
