# Supplementary figures and images for: Intratumoral and peritumoral radiomics for the pretreatment prediction of pathological complete response to neoadjuvant chemotherapy based on breast DCE-MRI
Source: Breast Cancer Res. 2017 May 18;19:57. doi: 10.1186/s13058-017-0846-1 (PMC5437672; doi:10.1186/s13058-017-0846-1)

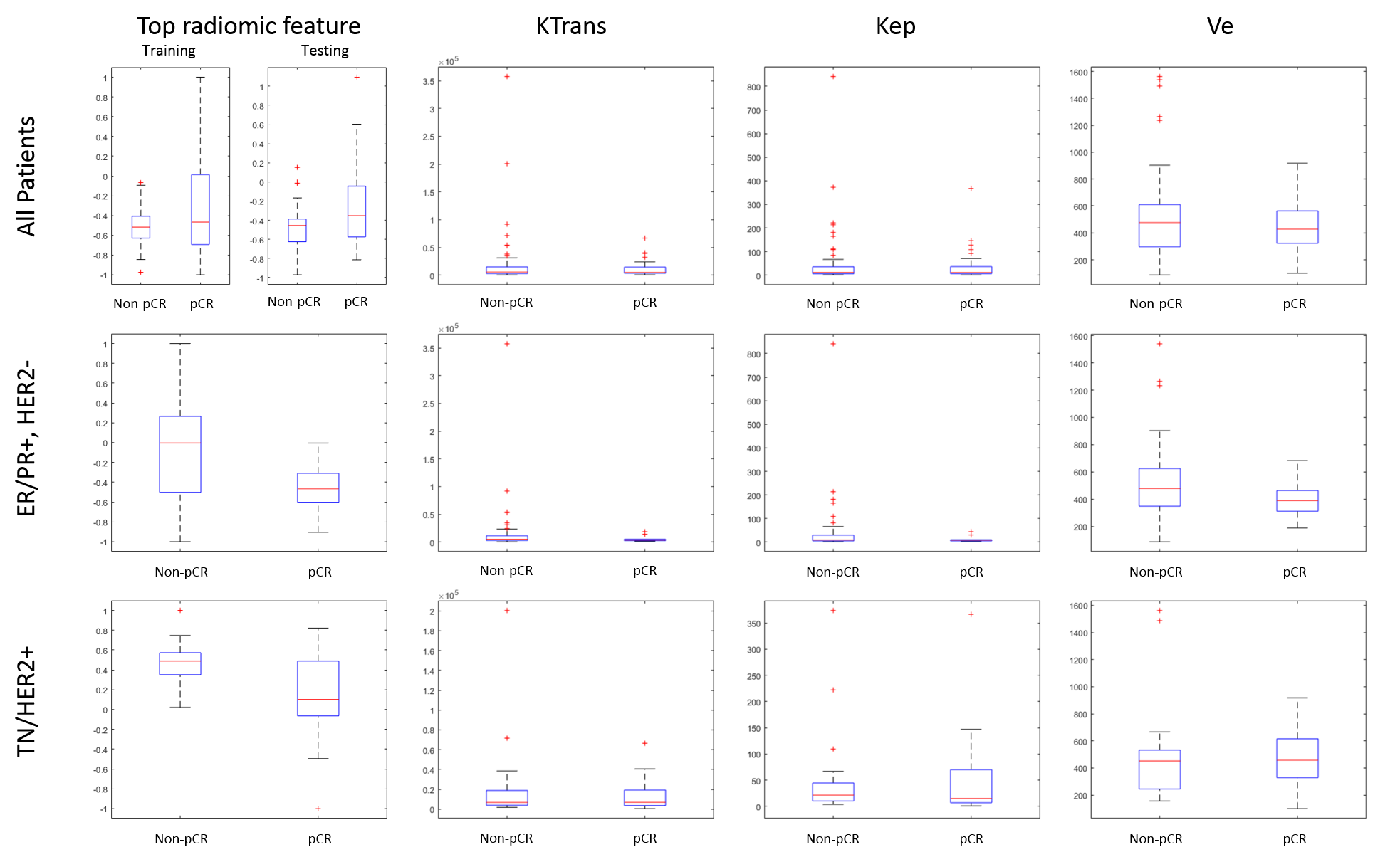

Supplement: Additional file 2: — Comparison of top radiomic and pharmacokinetic DCE-MRI parameters. Left: Box plots of top radiomic features among experimental groups. All-comers: kurtosis of intratumoral initial CoLlAGe information measure of correlation 1. HR+, HER2−: kurtosis of initial peritumoral CoLlAGe entropy. TN/HER2+: median of peritumoral peak Laws Level-Ripple. Right: Box plots of pharmacokinetic parameters (Ktrans, Kep, Ve) do not separate pCR and non-pCR among entire dataset, nor within HR+, HER2− and TN/HER2+ groups. (PNG 186 kb) [file 13058_2017_846_MOESM2_ESM.png]
