# Supplementary material for: Intratumoral and peritumoral radiomics for the pretreatment prediction of pathological complete response to neoadjuvant chemotherapy based on breast DCE-MRI
Source: Breast Cancer Res. 2017 May 18;19:57. doi: 10.1186/s13058-017-0846-1 (PMC5437672; doi:10.1186/s13058-017-0846-1)
Supplement: Additional file 3: Table S1. — Linear discriminant analysis (LDA) results for all experiments. Table S2. Diagonal linear discriminant analysis (DLDA) results for all experiments. Table S3. Support vector machine (SVM) results for all experiments. Table S4. Naïve Bayes results for all experiments. Table S5. Quadratic discriminant analysis (QDA) results for all experiments. (DOCX 35.9 kb) [file 13058_2017_846_MOESM3_ESM.docx]

Table S1. LDA results for all experiments.

Table S2. DLDA results for all experiments.

Table S3. SVM results for all experiments.

Table S4. Naïve Bayes results for all experiments.

Table S5. QDA results for all experiments.
