# Supplementary material for: Intratumoral and peritumoral radiomics for the pretreatment prediction of pathological complete response to neoadjuvant chemotherapy based on breast DCE-MRI
Source: Breast Cancer Res. 2017 May 18;19:57. doi: 10.1186/s13058-017-0846-1 (PMC5437672; doi:10.1186/s13058-017-0846-1)
Supplement: Additional file 4: — TN-specific and HER2+-specific response prediction. Table S6. Feature Discovery: HER2+ and TN tumors. Additional feature discovery experiments were performed for the subsets of HER2+ patients only (n=28) and TN patients only (n=19). Due to the limited sample size, only 5 top radiomic features were identified for each subtype. Table S7. Validation: HER2+ and TN tumors. The HER2+ and TN-specific signatures were evaluated by three-fold cross-validation of a DLDA classifier within the corresponding subset of patients and assessed by average AUC. Also shown are the results of using the top 5 features identified in the combined TN/HER2+ group for a DLDA classifier in a cross-validation setting within the combined TN/HER2+ group (n=47), HER2+ only group (n=28), and TN only group (n=19). (DOCX 17.2 kb) [file 13058_2017_846_MOESM4_ESM.docx]

| ***HER2+*** | *Feature Family* | *Descriptor* | *Location* | *Phase* | *Statistic* |
| --- | --- | --- | --- | --- | --- |
| 1 | Laws | Wave-Level | Peritumoral | Peak | Mean |
| 2 | Laws | Edge-Wave | Peritumoral | Peak | Median |
| 3 | Laws | Wave-Ripple | Peritumoral | Peak | Mean |
| 4 | Laws | Wave-Edge | Peritumoral | Peak | Skewness |
| 5 | CoLlAGe | Information Measure of Correlation 1 | Peritumoral | Peak | Skewness |
| ***TN*** | *Feature Family* | *Descriptor* | *Location* | *Phase* | *Statistic­­­* |
| 1 | Haralick | Sum Average | Intratumoral | Peak | Skewness |
| 2 | CoLlAGe | Difference Variance | Intratumoral | Peak | Mean |
| 3 | CoLlAGe | Sum Variance | Peritumoral | Initial | Skewness |
| 4 | Laws | Ripple-Spot | Intratumoral | Peak | Mean |
| 5 | Laws | Wave-Wave | Peritumoral | Peak | Mean |

Table S6. Feature Discovery: HER2+ and TN tumors. Additional feature discovery experiments were performed for the subsets of HER2+ patients only (n=28) and TN patients only (n=19). Due to the limited sample size, only 5 top radiomic features were identified for each subtype.

Table S7. Validation: HER2+ and TN tumors. The HER2+ and TN-specific signatures were evaluated by three-fold cross-validation of a DLDA classifier within the corresponding subset of patients and assessed by average AUC. Also shown are the results of using the top 5 features identified in the combined TN/HER2+ group for a DLDA classifier in a cross-validation setting within the combined TN/HER2+ group (n=47), HER2+ only group (n=28), and TN only group (n=19).
